# Stress distribution of complete-arch implant-supported prostheses reinforced with silica-nylon mesh

**DOI:** 10.4317/jced.56470

**Published:** 2019-12-01

**Authors:** Tarcisio-José de A. Paes-Junior, João-Paulo-Mendes Tribst, Amanda-Maria-de Oliveira Dal Piva, Marina Amaral, Alexandre-Luiz-Souto Borges, Fernanda-de-Cássia-Papaiz Gonçalves

**Affiliations:** 1 DDs, MSc, PhD, Adjunct Professor, Department of Dental Materials and Proshodontics, São Paulo State University (Unesp), Institute of Science and Technology, São José dos Campos / SP, Brazil; 2 DDs, MSc, PhD Student, Department of Dental Materials and Proshodontics, São Paulo State University (Unesp), Institute of Science and Technology, São José dos Campos / SP, Brazil; 3 DDs, MSc, PhD, Professor, Department of Dentistry (Prosthethic Dentistry), University of Taubaté (Unitau), Taubaté, Brazil; 4 DDs, MSc, PhD, Professor, Department of Prosthodontics, Brazcubas Education, Mogi das Cruzes, Brazil

## Abstract

**Background:**

This study evaluated the presence of a silica-nylon mesh and two cantilever lengths on the biomechanical behavior of complete-arch implant-supported prostheses.

**Material and Methods:**

Twenty-four (24) complete mandibular arch implant-supported prostheses were divided into 4 groups according to the presence of reinforcing mesh (with or without) and the cantilever length (molar – 15 mm or premolar – 5 mm). The specimens were submitted to strain gauge analysis (30-kgf, 10 s) at different points (molar and premolar). Three-dimensional models were created based on the in vitro specimens, and the results in the bone (microstrain), prostheses (tensile stress), implants and prosthetic screws (von-Mises stress) were evaluated using the finite element method (FEM). All materials were considered homogeneous, isotropic and linear. Strain gauge data were submitted to 3-way analysis of variance and the Tukey test (α=.05). FEM results were qualitatively analyzed using colorimetric graphs.

**Results:**

The microstrain magnitude for the prostheses with reinforcement was 519.91±359 and 583.33±661 without reinforcement (*p*=.001). The microstrain values for loading on the molar was 867.49±784 and on the premolar was 235.75±145. FEM corroborated with the in vitro findings for the bone behavior. The load application in the premolar showed reduced stress concentration, and a significant difference was observed between the presence or absence of the reinforcement for the prostheses.

**Conclusions:**

Silica-nylon mesh reduced the peri-implant microstrain and the prosthesis stress regardless of the cantilever extension. For temporary complete-arch implant-supported prostheses, the limitation of the cantilever to the premolar region improves the biomechanical response during load application.

** Key words:**Finite element analysis, biomechanical response, dental implants, prosthetic dentistry.

## Introduction

The material for manufacturing complete-arch implant-supported prostheses may influence the absorption and distribution of chewing loads on implants, and influence the strain/reabsorption of surrounding bone (1). The prosthetic planning, the passive adaptation of implant/prosthetic components, the number, distribution and position of implants in arch and the occlusal arrangement also have an influence on the stress distribution of the surrounding bone (2-4).

The incidence of excessive loads on the cantilever of an implant-supported prosthesis is a factor of extreme importance when the rehabilitation success is under evaluation. Excessive loading can lead to bone loss around the implant and to the prosthesis failure (3,5). In this way, the occlusal contacts must be adjusted so that the force is reduced in the cantilever region and distributed to the implants (6). However, it is not always possible to use a definitive prosthesis immediately, requiring the manufacture of a temporary structure to maintain the occlusion and masticatory function in the rehabilitated patient (7). In this sense, acrylic resin complete-arch implant-supported prostheses with provisional abutments provides a straight forward interim restoration with good tissue response (3,7). In a previous study it was demonstrated that incorporating silica-nylon mesh inside the acrylic resin of this prosthesis modality can increase the load bearing capacity during compression, regardless of the cantilever size (3). This result is justified since this silica-nylon mesh is able to increase the mechanical strength and dimensional stability of acrylic (8) and bis-acryl (9) resins. This mesh is composed of Nylon 6.0 (polyamide 6.0) and silanized silica (0.5% volume) in order to combine the favorable properties of both materials in a single body (3,9). Its manipulation allows it to be easily inserted inside the prosthesis and its flexibility allows this mesh to be individualized for each patient (3).

However, there are no data in the literature considering the effect of stress concentration on prostheses associated with nylon-silica mesh and adjacent structures such as implants and bone tissue. Therefore, the aim of this study was to investigate the biomechanical behavior *in vitro* and *in silico* of a complete-arch implant-supported prosthesis with and without silica-nylon mesh reinforcement and two cantilever lengths. The null hypothesis was that neither the silica-nylon reinforcement nor the cantilever length will affect the biomechanics behavior during posterior compressive load.

## Material and Methods

-Specimen preparation 

A replica of an edentulous jaw was made with polyurethane resin (F160; Axson Technologies). Five (5) equidistant perforations were made from a master implant-positioning model so that the final positions of the implants were parallel to each other and perpendicular to the horizontal plane of the polyurethane resin base, following the methodology applied by Gonçalvez *et al.* (2018) (3). To install five implants (3-mm exposure length) between the mental foramens, the sequence of spearhead, 2, 3, 3.15 and 3.5 milling surgery drills was used (Expertmatic E10C; KaVo do Brasil Ind Com Ltda.). The implants were placed with a stem vertical milling machine (B2; Bio-Art Equipamentos Odontológicos Ltda) to standardize the implant placement with a manual torque of 32 N.cm. The, micro conical abutments with a 2.5-mm metal strap were then installed on the implants with a torque of 20 N.cm (Conexão Sistemas de Próteses) (3). A polyvinylsiloxane (Elite HD+; ZhermackSpA) impression was performed and the implants’ position transferred in the mold. A stone model (Type IV gypsum cast, Zero Stone; Dentona) with implant analogs was obtained; the analog abutments were screwed to an titanium cylinder. The nylon mesh segment (BR 10.2012.028119.8) was positioned by alternating between the buccal and lingual sides of cylinders, as described in previous studies (3,8,9). After, the thermally activated acrylic resin was polymerized in a microwave oven (Continental AW-30; BS Continental da Amazônia Ltda) following the protocol of 900 W at 20% power for 20 minutes and 5 minutes at 60% power); after cooling, the prosthesis was finished with tungsten carbide burs (H251 EF; Komet) (3).

The groups were divided according to the presence of silica-nylon mesh (with or without) and the cantilever arm (premolar = 5 or molar = 15 mm): complete-arch implant-supported prosthesis without reinforcement and 5 mm cantilever; complete-arch implant-supported prosthesis without reinforcement and 15 mm cantilever; complete-arch implant-supported prosthesis with nylon reinforcement and 5 mm cantilever; and, complete-arch implant-supported prosthesis with nylon reinforcement and 15 mm cantilever (n=6) (3).

-*In vitro* strain measurement

**Figure 1:**
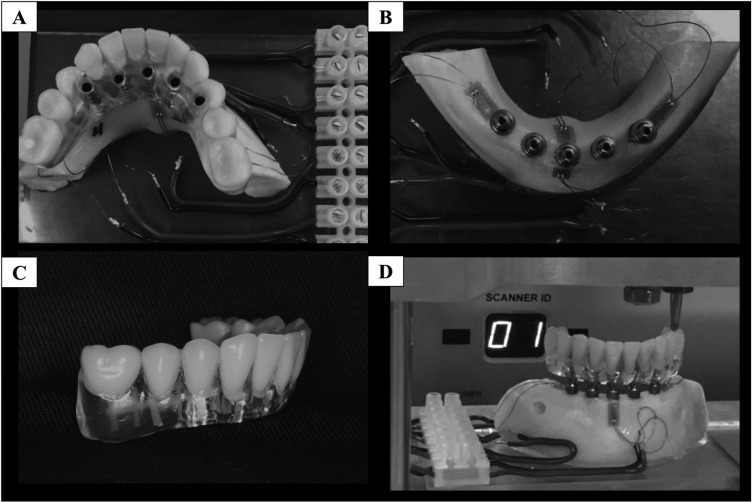
(A-D) - *In vitro* strain analysis. (A) Strain gauge positioning. (B) Occlusal and (C) lateral views of the *in vitro* specimen. (D) Load application. Strain gauge numbers were distributed as follows: 1 - distal to the most distal implant in the left; 2 - lingual region of the most anterior implant; 3 - buccal region of the most anterior implant; and 4 – distal to the most distal implant in the right.

The jaw (polyurethane) surface was polished with #220 sandpaper under water irrigation and cleaned with isopropyl alcohol. Four strain gauges (Excel - Excel Sensores Ind. Com. And Exportation Ltda.) with a length of 0.2 mm were glued 1mm below the implants, two of them were glued distally to the most distal implants, and the other two in the most anterior implant (vestibular and lingual) in superior view (Fig. 1) (10). The admeasurement of each strain gauge, 120 Ω, was performed using a multimeter (Minida ET 2055: Minida). The electrical connectors were bonded next to the jaw. The copper surface of the end plates and the two wires from each strain gauge were welded (Solderingunit: Mark VII Strain Gage Lineaccessories, Measurements Group Inc.; Solder connection: 44 rosin core solder) and were connected to an electrical signal conditioner (Model 5100 Scanner – System 5000 - Instruments Division Measurements Group, Inc.). The electrical variations are converted into units of microstrain (με) in the signal conditioner (10). After the strain gauge bonding, the prostheses were installed with 10 N.Cm in each prosthetic screw and submitted to 30 kgf loading (10) for 10 s in the left molar or premolar. These procedures were repeated two more times, totaling 3 loads per application point.

-*In silico* stress measurement

**Figure 2:**
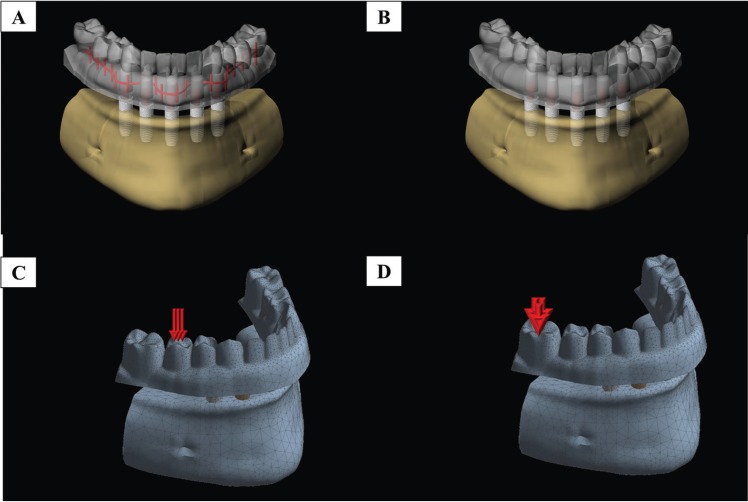
(A-D) - Schematic illustration of the modeling structures used in this study. Complete-arch implant-supported prosthesis (A) with and (B) without silica nylon mesh reinforcement and two loading areas (cantilever lengths): (C) premolar and (D) molar central fossa.

Identical groups used in the *in vitro* analysis were modelled and submitted to the finite element method to simulate the laboratory test and analyse the stress concentration in the prostheses and adjacent structures. One of the polyurethane jaws and both total prosthesis models (5 and 15 mm cantilever) were scanned (InEos, Sirona Dental Systems GmbH, Bensheim, Germany) and the three-dimensional files were obtained in stereolithographic format (.stl). Each file was then imported into the computer-aided design (CAD) software (Rinoceros 4.0, McNewel North America). Through a reverse engineering approach, the mesh surface was converted to a Nurbs surface, creating a solid and volumetric model (11) of the edentulous jaw and the prosthesis. The implants, their respective abutments and prosthetic screw models were then selected from the database of the Biomaterials and Biomechanics laboratory of the Institute of Science and Technology from São Paulo State University (Unesp/SJC), containing the same geometric specifications of the implants used in the *in vitro* test. Next, the implants were equidistantly distributed across the arch containing 3 mm of exposed threads similar to the *in vitro* model (3). The silica-nylon mesh was manually modeled in the CAD software containing 0.6 mm thick with 3 mm spacing between each fiber (8,9). Then, the prosthesis models were duplicated and a reinforcement was inserted in each region of the acrylic base. A Boolean difference was used to create the necessary space for the silica-nylon structure inside each prosthesis. Figure 2 summarizes the modeling structures and load application areas.

The 4 three-dimensional model files were exported to the analysis program (ANSYS 17.0) for the static structural mechanical analysis (10,11). After the mesh generation (following a mesh convergence test), all materials were considered homogeneous, isotropic and linear ( Table 1) (12). The elastic modulus of polyurethane and silica-nylon mesh were calculated by the excitatory pulse method. The contacts were considered perfectly bonded in all structures (10,11). The same load application (30 kgf) with vertical displacement restrictions of the base was performed according to the *in vitro* assay.

**Table 1:**
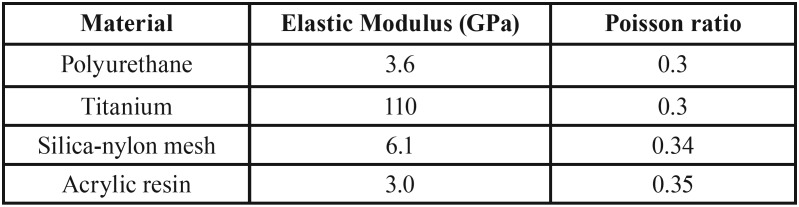
Material mechanical properties used in the computational analysis.

-Data analysis

Strain gauge analysis data were submitted to the statistical analysis using the Statistix computational program (Analytical Software inc., version 9.0, 2008), three-way analysis of variance (silica-nylon reinforcement, loading area and region of analysis) followed by post-hoc Tukey test (*p*=0.05). Finite element analysis results were plotted in colorimetric graphs and qualitatively analyzed. The stress peaks were selected for each structure for quantitative analysis. The stress values of the numerical model were assumed as valid since the mechanical behavior *in vitro* and *in silico* were similar for all groups.

## Results.

-*In vitro* strain measurement

Three-way ANOVA showed that each isolated factor affected the microstrain values: silica-nylon mesh presence with F = 23.90 and *P* < 0.001; load application with F = 2,372.35 and *p* < 0.001; and the region of analysis (strain gauge position) with F = 1,505.30 and *p* < 0.001. The prostheses with silica-nylon mesh (519.91±359)B showed lower microstrain values than without (583.33±661)A it. The loading on the molar (867.49±784)A showed higher mean values than on the premolar (235.75±145)B. The interaction of silica-nylon mesh*strain gauge position showed statistical significance with F = 3.16 and *p* = 0.027. The strain gauge nearest the implant under load application showed the highest value of bone microstrain, and the prosthesis containing the silica-nylon mesh significantly decreased this value (1365 to 1233). The same behavior occurred for all implants at a lower magnitude.

-*In silico* stress measurement

**Figure 3:**
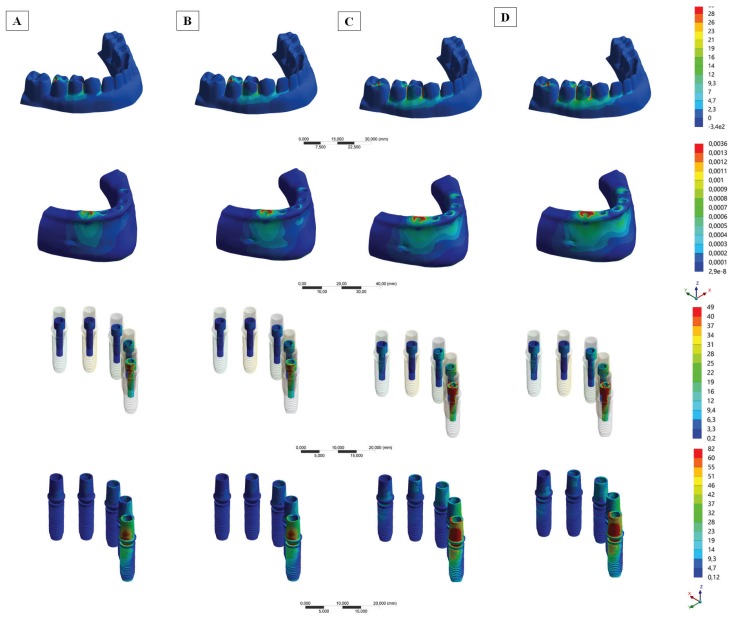
(A-D) Tensile stress (maximum principal stress) results in the prostheses,Microstrain results in the bone, Von-Mises stress results in the implants and prosthethic screws according to the silica-nylon reinforcement and loading area. Complete-arch implant-supported prosthesis (A and C) with and (B and D) without silica nylon mesh reinforcement and two loading areas (cantilever lengths): (A and B) premolar central fossa and (C and D) molar central fossa.

In observing the results of the *in silico* assay in the bone tissue (Fig. 3), it is possible to observe that the load application in the molar region presented qualitatively more stress around the last implant than the application of load in the premolar region. For the prosthesis (Fig. 3), the generated tensile stress demonstrated that a possible failure could occur between the tooth that received the load application and the next mesial tooth. Also, that the load applied in the premolar resulted in lower stress concentration. Likewise, the presence of the silica-nylon mesh inside the prosthesis decreased the generated stress, but with a lower effect than the loading area factor. The most stressed implants and prosthetic screws (Fig. 3) were those close to the loading site, and the effect of reducing the generated stress when the load is applied in a smaller cantilever (premolar) is visible. It was not possible to observe the influence of the silica-nylon mesh in these structures.

## Discussion

The results of the present study demonstrated that the presence of the silica-nylon mesh inside the prosthesis is significant to change the mechanical response of the system, reducing the microstrain and stress values in the peri-implant tissue regardless of the cantilever extension (loading area). Thus, rejecting the null hypothesis.

It is reported that the main failure causes of osseointegrated implants are related to biomechanical factors (14). Therefore, the present study demonstrated that it is possible to improve the biomechanical response using a properly constructed prosthesis and smaller cantilever.

The literature reports that the use of cantilevers in supported implants should be restricted to the maximum limit of 15 to 20 mm (15). This restriction in cantilever length occurs because it can increase the occlusal forces transmitted to the bone tissue (16). Our results corroborated those findings since the load application region was significant, with the molar region being more damaging for the supporting tissue than the premolar region.

It is well-known that the bone quality for installing implants is of extreme importance. The bone adjacent to the implants is composed of trabeculae and lamellae varying in size and number between regions, varying according to the patient’s age, functional status and systemic factors (17), which makes it difficult to standardize an experimental model. In order to facilitate the confection and reproduction of the experimental model, several *in vitro* studies (2,4) replace the bone tissue using homogeneous and isotropic materials such as polyurethane. However, the results presented herein should be carefully extrapolated and supplemented with other studies, since bone tissue has particularities that are not reproduced in this resin.

The mechanical stimuli in the bone tissue must be above 100 microstrain in order to avoid bone remodeling for disuse (18). In addition, these stimulus values cannot exceed the physiological limit (3000) as this would also lead to reabsorption (19). In this sense, none of the models evaluated in this study had potential to induce bone resorption, either due to disuse or because it exceeded the physiological limit. However, the loading in the molar (15 mm cantilever) does not seem to be indicated since the maximum calculated values were greater than 2000 με in the strain gauge region distal to the posterior implant near the loading site ( Table 2). Close to 10 times lower microstrain values were calculated when the loading occurred in the premolar region. Thus, a safety factor for the clinician would be to use the silica-nylon mesh and reduce the size of the prosthesis cantilever (3). These results corroborated with *in silico* (2) *in vitro* (20) and *in vivo* (21) studies which observed the largest microstrain values occurring near the load application point, indicating that the produced stresses around the implants are dependent on the loading site. In addition, both load and the presence of the mesh reinforcement influenced the mechanical response of the numerical model, corroborating with the behavior observed *in vitro*. This allows us to assume the model as valid and quantitatively observe the stress concentration regions which cannot be observed with the strain gauge method.

**Table 2:**
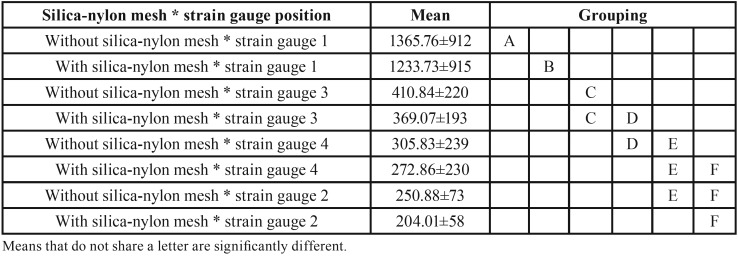
Mean microstrain values (με), standard deviation and post-hoc Tukey test (α=5%) for the “silica-nylon reinforcement*strain gauge position” Interaction.

In a retrospective cohort study evaluating 114 patients who were treated with complete-arch implant-supported prosthesis, 54.39% of the patients experienced prosthetic complications over 6 months. The authors concluded that fiber-reinforcement could reduce the prevalence of resin base fractures in acrylic immediate prosthesis (22). This is completely in accordance with the present study which showed an improvement in the mechanical response with the use of silica-nylon mesh reinforcement for this prosthesis modality. In a prospective cohort study, a previous paper evaluated 18 patients restored with implant-supported fiber-reinforced resin prostheses (23). The authors did not find prosthetic failure, chipping or fracture within the first year of loading. Thus, the use of fiber-reinforced prosthesis should be promising for full-arch rehabilitation, but the silica-nylon mesh presented herein could reduce the treatment costs for the patient even more.

As limitations of this study, it is possible to consider that the in vitro test only evaluated the microstrain in four regions. The computational method was used to complement this test, however this method also has limitations. The 3D model used in this study did not consider anatomical variations and did not simulate oral medium conditions which involves, for example, temperature variation, parafunctional habits and different masticatory load values which could modify the results.

The following conclusions can be made based on the results:

1. Silica-nylon mesh reduced the peri-implant microstrain and the prosthesis stress, regardless of the cantilever extension.

2. For temporary complete-arch implant-supported prostheses, the limitation of the cantilever to the premolar (5 mm) improves the biomechanical response during load application.
